# Rapamycin Alleviates Protein Aggregates, Reduces Neuroinflammation, and Rescues Demyelination in Globoid Cell Leukodystrophy

**DOI:** 10.3390/cells12070993

**Published:** 2023-03-24

**Authors:** Dar-Shong Lin, Yu-Wen Huang, Tsung-Han Lee, Lung Chang, Zon-Darr Huang, Tsu-Yen Wu, Tuan-Jen Wang, Che-Sheng Ho

**Affiliations:** 1 Department of Pediatrics, MacKay Memorial Hospital, Taipei 10449, Taiwan; 2 Department of Medicine, MacKay Medical College, New Taipei 25245, Taiwan; 3 Department of Medical Research, MacKay Memorial Hospital, Taipei 10449, Taiwan; 4 Department of Laboratory Medicine, MacKay Memorial Hospital, Taipei 10449, Taiwan; 5 Department of Neurology, MacKay Children’s Hospital, Taipei 10449, Taiwan

**Keywords:** psychosine, globoid cell leukodystrophy, Krabbe disease, demyelination, autophagy, ubiquitin-proteasome system, neuroinflammation

## Abstract

We have shown in vivo and in vitro previously that psychosine causes dysfunction of autophagy and the ubiquitin-proteasome system underlying the pathogenesis of globoid cell leukodystrophy (GLD), a devastating lysosomal storage disease complicated by global demyelination. Here, we investigated the therapeutic efficacy of the mTOR inhibitor rapamycin in twitcher mice, a murine model of infantile GLD, in biochemical, histochemical, and clinical aspects. Administration of rapamycin to twitcher mice inhibited mTOR signaling in the brains, and significantly reduced the accumulation of insoluble ubiquitinated protein and the formation of ubiquitin aggregates. The astrocytes and microglia reactivity were attenuated in that reactive astrocytes, ameboid microglia, and globoid cells were reduced in the brains of rapamycin-treated twitcher mice. Furthermore, rapamycin improved the cortical myelination, neurite density, and rescued the network complexity in the cortex of twitcher mice. The therapeutic action of rapamycin on the pathology of the twitcher mice’s brains prolonged the longevity of treated twitcher mice. Overall, these findings validate the therapeutic efficacy of rapamycin and highlight enhancing degradation of aggregates as a therapeutic strategy to modulate neuroinflammation, demyelination, and disease progression of GLD and other leukodystrophies associated with intracellular aggregates.

## 1. Introduction

Globoid cell leukodystrophy (GLD), commonly referred to as Krabbe disease, is a devastating lysosomal storage disease (LSD) complicated with global demyelination as nearly 90% of GLD patients present in the most severe infantile form associated with profound morbidity and mortality [1,2]. Early-onset GLD infants present symptoms as early as 2 months of age with irritability and hypotonia, followed by pyramidal signs, seizures, tonic spasms, rapid regression to incapacitation, and death by 2 years of age. It is caused by autosomal recessive mutations of gene encoding lysosomal enzyme galactocerebrosidase (GALC), which catalyzes the initial step in the degradation of galactosyl-ceramide during the process of myelin synthesis [3,4]. Deficiency of GALC activity results in impairment of myelin synthesis and accumulation of cytotoxic metabolite psychosine, which causes the death of oligodendrocytes leading to global demyelination in both central (CNS) and peripheral nervous systems (PNS) [5,6,7]. To study the pathological mechanism and interventions of GLD, an authentic murine model, twitcher mice, was widely used in studies. Twitcher mice, harboring naturally occurred Galc mutations, develop an accumulation of psychosine, massive neuro-inflammation, broad demyelination in the nervous system, and premature death around 40 days of age, recapitulating the pathophysiology of human GLD [8].

The observation that accumulation of psychosine in CNS and PNS is responsible for the clinical symptoms associated with GLD, leads to the proposal of the “psychosine hypothesis”, and forms the mechanistic basis for the pathogenesis of GLD [9]. It has been recently confirmed that GALC-deficient mice concomitant with the elimination of psychosine accumulation do not manifest with GLD pathogenesis [10]. Meanwhile, both in vivo and in vitro studies demonstrate that the aberrant accumulation of endogenous psychosine in oligodendrocytes affects their differentiation and maturation leading to apoptotic cell death and impaired myelination [11]. Additionally, progressive accumulation of psychosine inhibits antegrade and retrograde fast axonal transport and initiates an axonal dying-back degeneration and neuronal vulnerability [12]. These psychosine-mediated alternations of cellular functions elicit a myriad of pathogenic events, including demyelination and neurodegeneration, within GLD brains. Besides these pathologic changes, robust recruitment and accumulation of reactive microglia, globoid cells, and astrocytes are observed in demyelination within the CNS of GLD-affected humans and animal models [13]. Collectively, all these pathogenic events lead to remarkable neuroinflammation concomitant with broad demyelination within GLD brains at end stage, as pathological hallmarks of GLD.

Recently, our in vivo and in vitro studies described the psychosine induced impairments of the ubiquitin-proteasome system (UPS) and autophagy that underlie the pathological mechanism of GLD [14]. Autophagy and UPS are the two major intracellular degradation systems that maintain cellular protein homeostasis [15]. Dysfunction of autophagy has been observed in the brains of twitcher mice and both human and twitcher mice-derived oligodendrocytes and fibroblasts under treatment of psychosine [16,17,18,19]. It is noted that elevation of cleaved microtubule-associated protein light-chain 3 (LC3)-II and p62/sequestosome 1 (p62), and the accumulation of autolysosomes in psychosine-treated oligodendrocytes signal a disruption of the autophagy pathway [14,17]. The deposition of p62 aggregates in the brain of twitcher mice further validates the inability to degrade misfolded proteins by the autophagy pathway [14,16]. Our previous studies further showed that both the accumulation of insoluble ubiquitin and the deposition of ubiquitin aggregates occur in the brains of twitcher mice and oligodendrocytes under treatment of psychosine, and involve the dysfunction of UPS in the degradation of misfolded and damaged proteins [14]. These findings highlight the psychosine-driven vulnerability of misfolded and damaged proteins contributing to the pathogenesis of GLD. At the same time, these findings provide the rationales for investigating whether increasing clearance of disease protein might provide a benefit to GLD.

Autophagy is negatively regulated by the mechanistic target of the rapamycin (mTOR) signaling pathway, which is specifically inhibited by the macrolide antibiotic rapamycin in various cell types and model systems [20]. Furthermore, it has been demonstrated that inhibition of mTOR by rapamycin upregulates autophagy as well as UPS to increase overall protein degradation [21]. Currently, rapamycin and its analogs are FDA-approved antibiotics used for the treatment of tuberous sclerosis complex, cancer, preclinical studies for cancer, cell growth and metabolism, neurodegeneration, and aging [22]. Upregulation of proteolytic degradation by treatment of rapamycin has been shown to accelerate the clearance of misfolded proteins leading to amelioration of neurodegeneration in murine models of spinobulbar muscular atrophy and Huntington’s disease [23,24,25]. In this study, we determine the therapeutic effect of rapamycin in twitcher mice, the murine model of GLD, and report a reduction of protein aggregates, alleviation of neuroinflammation and demyelination, preservation of myelin integrity, and prolongation of lifespan.

## 2. Materials and Methods

### 2.1. Animals

The colonies of heterozygous (twi/+) twitcher mice with C57BL/6J genetic background obtained from the Jackson Laboratory (Bar Harbor, ME) were inbred and maintained in a pathogen-free environment in the animal research facility of MacKay Memorial Hospital. The twitcher mice were genotyped at 3 days of age using a molecular PCR assay as previously described [26]. Wild-type (+/+) and homozygous (twi/twi) animals were used for the experiments. For observation of phenotype, the experimental mice were allowed to live freely until the moribund stage. For biochemical and histochemical studies, the experimental mice at day 35 were euthanized under anesthesia with pentobarbital and the brains were rapidly removed and dissected into two sagittal parts. One hemisphere was snapped frozen with liquid nitrogen and stored at −80 °C before biochemical analysis, and another hemisphere was fixed in formalin for 24 h and embedded in paraffin for immunohistochemistry. All animal procedures were performed following guidelines for the care of animals approved by the Animal Care and Use Committee of MacKay Memorial Hospital.

### 2.2. Immunofluorescent Staining

Sections of paraffin-embedded brain tissues (4 μm) were baked for 1 h at 65 °C, de-waxed in xylene, rehydrated in a serial concentration of ethanol, and immersed in phosphate buffered saline (PBS) buffer. The sections were boiled with Tris-EDTA buffer (pH 9) in a high-pressure cooker for 10 min, cooled to room temperature, washed with PBS buffer, and permeabilized using 0.5% Triton X-100 in PBS for 10 min. After blocked in 5% horse serum/0.25% Triton X-100 in PBS for 30 min at room temperature, sections were incubated with primary antibodies over night at room temperature. Primary antibodies used were mouse anti-p62 (Abcam, Cambridge, UK), mouse anti-Ubiquitin (Abcam), mouse anti-Myelin basic protein (MBP; Millipore, Burlington, MA, USA), mouse anti-Glial fibrillary acidic protein (GFAP; Invitrogen, Eugene, OR, USA), rabbit anti-GFAP (Invitrogen), mouse anti-proteolipid protein (PLP; Abcam), rabbit anti-PLP (Abcam), and rabbit anti-ionized calcium-binding adapter molecule 1 (Iba1; Wako, Osaka, Japan). After washing with PBS buffer, the sections were incubated with Alexa Four 488- and Alexa Four 594-conjugated secondary antibodies (Invitrogen) for 4 h at room temperature, washed with PBS buffer, counter stained with DAPI for 5 min, and cover-slipped. The fluorescence was visualized under the Leica DM IL LED immunofluorescence microscope and the images exported for further analysis.

### 2.3. Image Analysis

Quantitative assessment of p62- and ubiquitin-aggregates, GFAP-positive astrocytes, and Iba1-positive microglia were analyzed using Fiji package of ImageJ software (National Institutes of Health, Bethesda, MD, USA). The total particle counts of p62- and ubiquitin-aggregates, cell counts of astrocytes and microglia in the brain stem region were measured and calculated as counts /mm^2^. The total surface area of p62- and ubiquitin-aggregates, astrocytes, and microglia in the brain stem region was measured and calculated as μm^2^/mm^2^.

GFAP-positive astrocytes and Iba1-positive microglia were readily identified, classified, and quantified according to their cellular morphology. Accordingly, morphological characteristics of microglia were classified from resting (small round or slender soma, ramified processes), activated (hypertrophic soma, bushy and thick processes), to reactive (amoeboid, multinucleated) cells [27,28]. Morphologically, astrocytes were classified as resting (small round or slender soma with thin processes) and reactive (hypertrophic soma with numerous thicker and extended processes) cells [28]. We calculate the total number and surface area of astrocytes and microglia cells/mm^2^ in brain stem and surface area per astrocyte and microglia.

To determine the level of myelination, we measured the proportion of myelinated cortex and density of myelin fibers in the cortex of MBP-labelled images as previously described [29]. The MBP-labelled immunofluorescent images of cortex above body of corpus callosum were converted to gray images, and the percentage of myelinated cortex was calculated as follows: (myelinated cortex/total cortex) × 100%. The gray images were converted to binary images after image segmentation and removal of the background. The density of myelinated neurites in cortex was calculated as follows: (neurites area/cortex area). The microstructural complexity of cortical myelination was quantitatively analyzed by counting the number of intersections per concentric shell with radii increase of 10 μm using the Sholl analysis function of “ImageJ” plugin. The total number of neurites intersections in the cortical field was expressed as intersections counts/mm^2^.

### 2.4. Western Blot Analysis

The detergent soluble and insoluble fractions of protein from brain tissues for western blotting were extracted and separated as previously described [14]. In brief, brain tissue was homogenized and lysed with T-PER™ Tissue Protein Extraction Reagent (Thermo Fisher Scientific, Eugene, OR, USA) supplemented with Halt^TM^ protease inhibitor cocktail (Thermo Fisher Scientific). After centrifugation at 17,000× *g* for 20 min at 4 °C, the collected supernatant was labeled as the detergent soluble fraction. While insoluble pellet was resuspended in T-PER™ Tissue Protein Extraction Reagent supplemented with 2% sodium dodecyl sulfate (SDS; Millipore) and sonicated on ice. The supernatant collected after centrifugation at 17,000× *g* for 20 min at 4 °C was labeled as insoluble fraction. Protein concentration was determined with BCA protein assay (Thermo Fisher Scientific) and an aliquot of protein was denatured. Electrophoresis was performed on 10~12% SDS-polyacrylamide gel, followed by a transfer to a PVDF membrane (Millipore), which was blocked with 0.5% nonfat milk in TBST [20 mM Tris-HCl (pH 7.5), 150 mM NaCl, 0.1% Tween 20] and incubated with primary antibodies overnight at 4 °C. Primary antibodies used were rabbit anti-LC3 (Sigma, St Louis, MO, USA), mouse anti-p62 (Abcam), rabbit anti-Ubiquitin (Cell Signaling), rabbit anti-S6 ribosomal protein (Cell Signaling, Danvers, MA, USA), rabbit anti-phospho-S6 Ribosomal protein (Cell Signaling), mouse anti-MBP (Millipore), mouse anti-GFAP (Invitrogen), mouse anti-beta-actin (Sigma) and mouse anti-Histon-3 (H3; Cell Signaling). Membranes were incubated with horseradish peroxidase (HRP)-conjugated secondary antibody and developed with Immobilon Western Chemiluminescent HRP Substrate (Millipore) at room temperature. The chemiluminescent signal was obtained by ChemiDoc-It 815 Imaging System (Analytik Jena US LLC, Upland, CA, USA).

### 2.5. Weight Gain and Longevity

The untreated twitcher mice died at around 38 to 42 days of age. Commencing on day 21, twitcher mice were injected intraperitoneally with rapamycin 3 mg/kg/day every other day for three doses per week and allowed to live freely until the moribund stage. The mice were checked daily and weighed on a weekly basis. The untreated twitcher and wild-type mice will be identified and used as control animals to determine their life span. Kaplan–Meier analysis will be used to compare the life spans of these groups.

### 2.6. Statistical Analysis

All results were expressed as the mean ± SD or mean ± SEM. Statistical tests were performed using GraphPad Prism 6.0 software package. One-way ANOVA Tukey post-hoc test was used for multiple comparisons among three groups and Student’s *t*-test was used for comparing between two groups, with *p*-values less than 0.05 as statistical significance.

## 3. Results

### 3.1. Rapamycin Upregulates Autophagy

**Figure 1:**
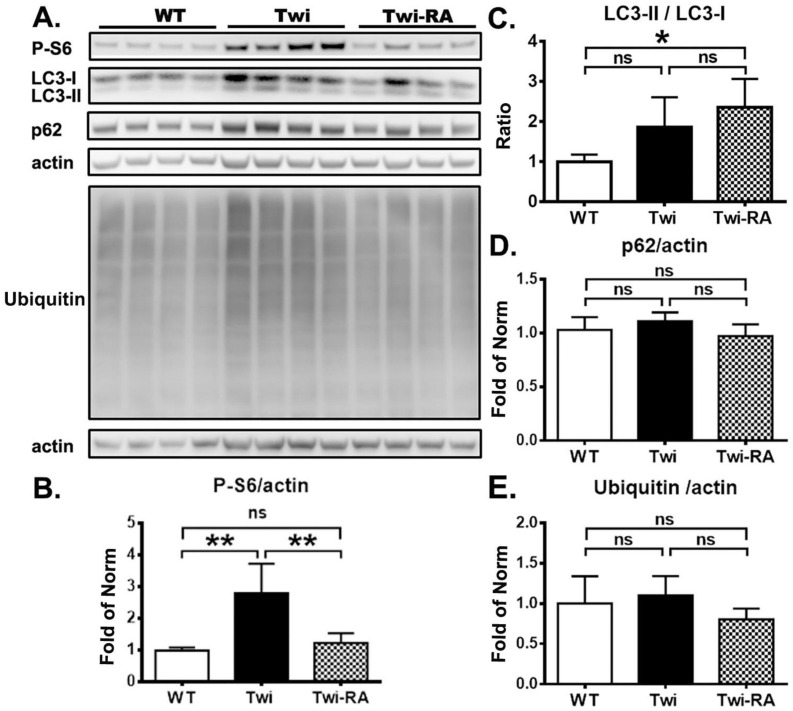
Rapamycin upregulates autophagy. (**A**) Proteins were extracted from Triton-X buffer (soluble fraction). Immunoblotting analyzed for autophagy related proteins phospho-S6 (P-S6), LC3-I, LC3-II, sequestosome1/p62 (p62), and ubiquitin in brain stem from wild-type (WT), untreated twitcher (Twi), and rapamycin-treated twitcher (Twi-RA) mice at 35 days of age. Quantitative analysis of the results of immunoblots are shown in (**B**) for P-S6, in (**C**) for LC3 II/LC3-I ratio, in (**D**) for p62, and in (**E**) for ubiquitin. Protein levels are normalized to actin. Values are expressed as mean ± SD and compared using One way ANOVA (Tukey post hoc test) (*n* = 4). Statistical significance, * *p* < 0.05, ** *p* < 0.01, and ns (not significant).

In the present study, we explored the therapeutic efficacy of rapamycin upon demyelination in twitcher mice. A dose of rapamycin at 3 mg/kg/day was injected intraperitoneally every other day for 3 doses per week commencing on day 21. Rapamycin is a canonical inhibitor of mTOR complex 1 (mTORC1), which regulates autophagy through direct phosphorylation of ribosomal protein *S6* kinase 1 (S6). Previous studies indicated that axonal damages and deposition of aggregates were most profound in the brain stem [14,30]. Herein, the levels of phosphorylated-S6 (P-S6), LC3, p62, and ubiquitin in brain stem homogenates (*n* = 4 in each group) were determined by western blotting as measures of activation of the mTOR pathway, autophagy, and UPS, respectively (Figure 1A). It was shown that the brain stems of untreated twitcher mice demonstrated a significant increase of P-S6 expression compared to wild-type mice (Figure 1B). In contrast to this, administration of rapamycin to twitcher mice reduced the expression of P-S6 significantly compared to untreated twitcher mice, indicating the inhibition of mTORC1. The level of P-S6 expression in rapamycin-treated twitcher mice was at a moderately higher level than that of wild-type mice, albeit without significant difference. This result validates the pharmacological effect of rapamycin in twitcher mice.

Among the molecules participating in the process of autophagy, p62 facilitating selective degradation and the conversion of LC3-I to LC3-II via phosphatidylethanolamine conjugation during autophagosome formation have been used as markers for assessment of autophagic flux [15]. To further determine the inhibition effects of mTORC1 by rapamycin in the activation of autophagy, the ratio of LC3-II/LC3-I and levels of p62 were measured in a soluble fraction of brain homogenates. Accordingly, there was no statistically significant difference in ratio of LC3-II/LC3-I in the soluble fraction of brain stem homogenates between untreated twitcher mice and wild-type mice (Figure 1C). The ratio of LC3-II/LC3-I in rapamycin treated twitcher mice was indistinguishable from that of untreated twitcher mice, but was elevated significantly in comparison to that of wild-type mice, indicating an increased autophagic flux by rapamycin (Figure 1C).

Furthermore, we observed no significant difference in the level of soluble p62 between untreated twitcher mice and wild-type mice (Figure 1D). The administration of rapamycin to twitcher mice did not significantly alter the p62 expression compared to both untreated twitcher mice and wild-type mice (Figure 1C). Given that p62 links the autophagy pathway and the UPS upon ubiquitinated protein degradation, the level of ubiquitin was determined accordingly. The levels of soluble ubiquitin between wild-type, untreated- and rapamycin-treated twitcher mice did not show statistically significant differences (Figure 1E).

**Figure 2:**
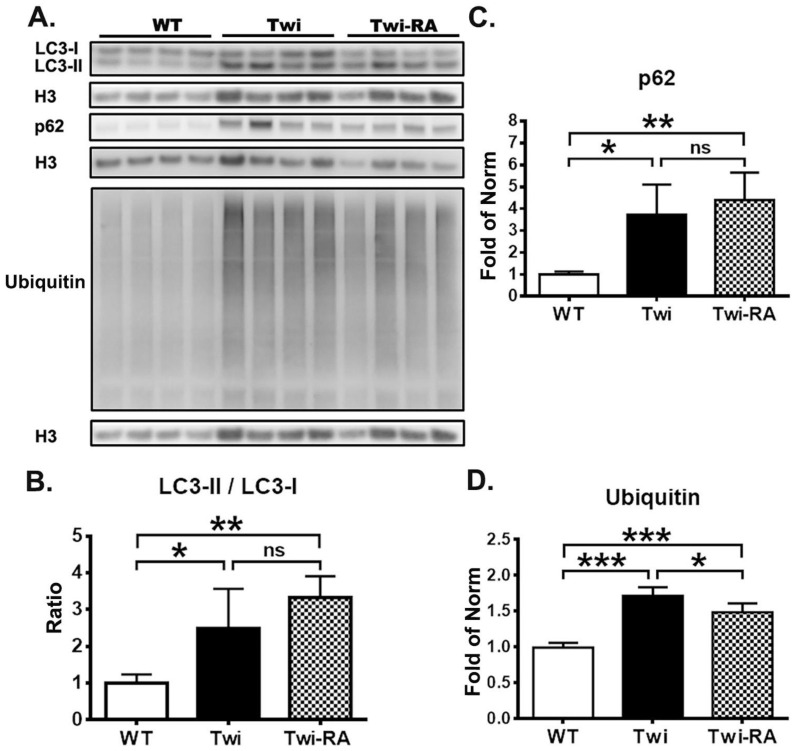
Rapamycin decreases levels of insoluble ubiquitin. (**A**) Insoluble proteins were extracted from SDS buffer and probed for autophagy related proteins LC3-I, LC3-II, p62, and ubiquitin in brain stems from wild-type (WT), untreated twitcher (Twi), and rapamycin-treated twitcher (Twi-RA) mice at 35 days of age. Quantitative analysis of the results of immunoblots are shown in (**B**) for LC3 II/LC3-I ratio, in (**C**) for p62, and in (**D**) for ubiquitin. Protein levels are normalized to Histon-3 (H3) for insoluble fraction. Values are expressed as mean ± SD and compared using One way ANOVA (Tukey post hoc test) (*n* = 4). Statistical significance, * *p* < 0.05, ** *p* < 0.01, *** *p* < 0.001, and ns (not significant).

Our previous studies showed an impairment of both autophagy and UPS leading to accumulation of insoluble p62 and ubiquitin in twitcher mice [14]. Accordingly, we measured the levels of LC3, p62, and ubiquitin in the insoluble phase of brain homogenates from wild-type mice, twitcher mice, and rapamycin-treated twitcher mice (*n* = 4 in each group). Under our previous studies in the insoluble phase of brain homogenates, LC3-II/LC3-I ratio in twitcher mice was elevated significantly compared to wild-type mice (Figure 2B). The insoluble phase LC3-II/LC3-I ratio in rapamycin-treated twitcher mice was indistinguishable with that of untreated-twitcher mice, but this ratio was elevated significantly in comparison with that of wild-type mice (Figure 2B). In line with our previous studies, the levels of insoluble p62 and ubiquitin in untreated twitcher mice were elevated significantly compared to wild-type mice (Figure 2C,D). Rapamycin treated twitcher mice also showed a significant increase in both insoluble p62 and ubiquitin expression compared to wild-type mice. Nonetheless, administration of rapamycin to twitcher mice reduced the accumulation of insoluble ubiquitin significantly compared to untreated twitcher mice (Figure 2D).

Overall, in accordance with previous studies, these findings support the dysregulation of autophagy and UPS, leading to accumulation of insoluble p62 and ubiquitin in twitcher mice. Furthermore, rapamycin upregulates the autophagic flux and decreases the accumulation of insoluble ubiquitin.

### 3.2. Reduction of Ubiquitin- and p62-Aggregates

**Figure 3:**
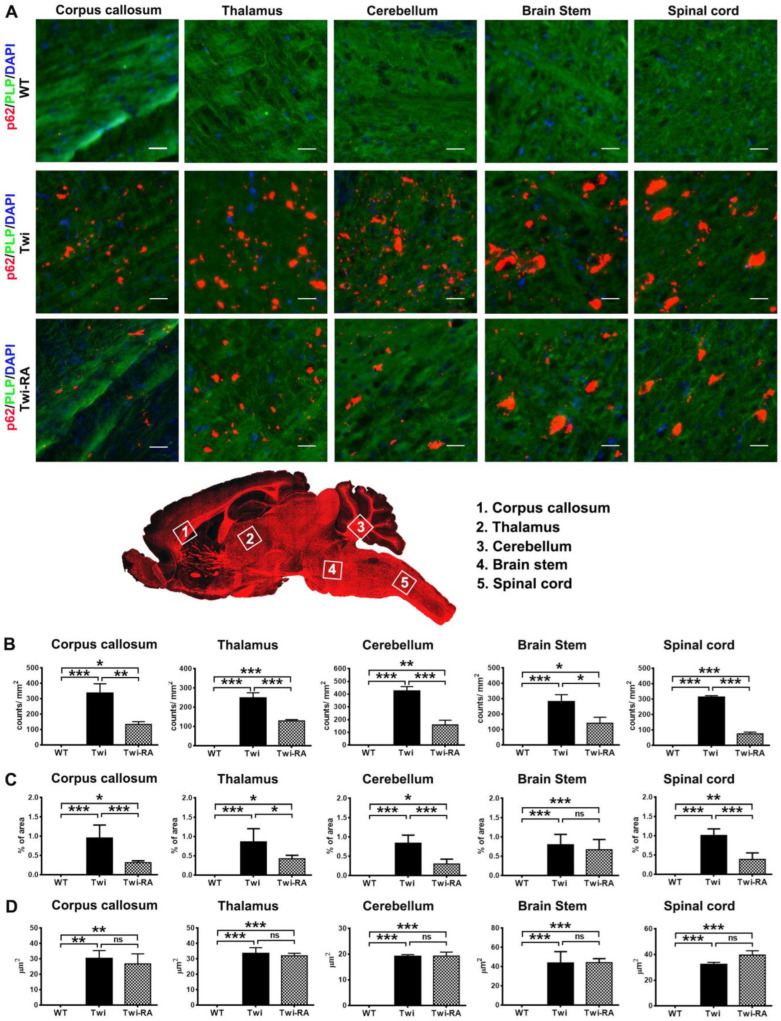
Total particles of p62 aggregates alleviated by rapamycin. (**A**) Representative images of p62 (red), PLP (green), and nuclei (DAPI) immunostaining in different regions of the brain of wild-type (WT), untreated twitcher (Twi), and rapamycin-treated twitcher (Twi-RA) mice at 35 days of age. Scale bars: 200 μm. Total amounts of p62 aggregates per square millimeters (**B**), the percentage of area occupied by p62 aggregates (**C**), and the average size of p62 aggregates (**D**) were quantitatively analyzed with ImageJ. Values are expressed as mean ± SEM and compared using One way ANOVA (Tukey post hoc test) (*n* = 5). Statistical significance, * *p* < 0.05, ** *p* < 0.01, *** *p* < 0.001, and ns (not significant).

The insoluble p62 and ubiquitin were not degraded by impaired autophagy and UPS, thus they accumulated intracellularly leading to the formation of intracellular aggregates in the brains of twitcher mice along with the progression of the disease [14]. In the present study, western blot analysis detected insoluble proteins which need to be degraded, while immunofluorescent staining demonstrated the deposition of aggregates which cannot be degraded and are cytotoxic to oligodendrocytes. Using antibodies directed against ubiquitin and p62, both the distribution of p62 and ubiquitin aggregates in the brains of experimental animals (*n* = 5 in each group) were illustrated by immunofluorescent staining. In line with our previous studies, p62 aggregates were distributed broadly in white matters and were abundant in the spinal cord, brain stem, cerebellum arbor vitae, thalamus, and corpus callosum of twitcher mice (Figure 3A) [14]. Rapamycin-treated twitcher mice showed similar distribution with decreased expression of p62 aggregates in the brain compared to untreated twitcher mice using qualitative assessment. With quantitative analysis by ImageJ, total particle numbers and surface area of p62 aggregates in the brain stem were further compared between groups. As expected, particles of p62 aggregates were abundant in untreated twitcher mice, while the administration of rapamycin to twitcher mice significantly reduced the total number of particles of p62 aggregates to nearly 50% of that of untreated twitcher mice (Figure 3B). Furthermore, the percentage of area occupied by p62 aggregates was significantly reduced in the corpus callosum, thalamus, cerebellum, and spinal cord of rapamycin-treated twitcher mice in comparison with that of untreated twitcher mice (Figure 3C), albeit there was no statistical significance in average size of p62 aggregates (Figure 3D). Overall, administration of rapamycin to twitcher mice reduced the total number of particles of p62 aggregates and the surface area of p62 aggregates.

**Figure 4:**
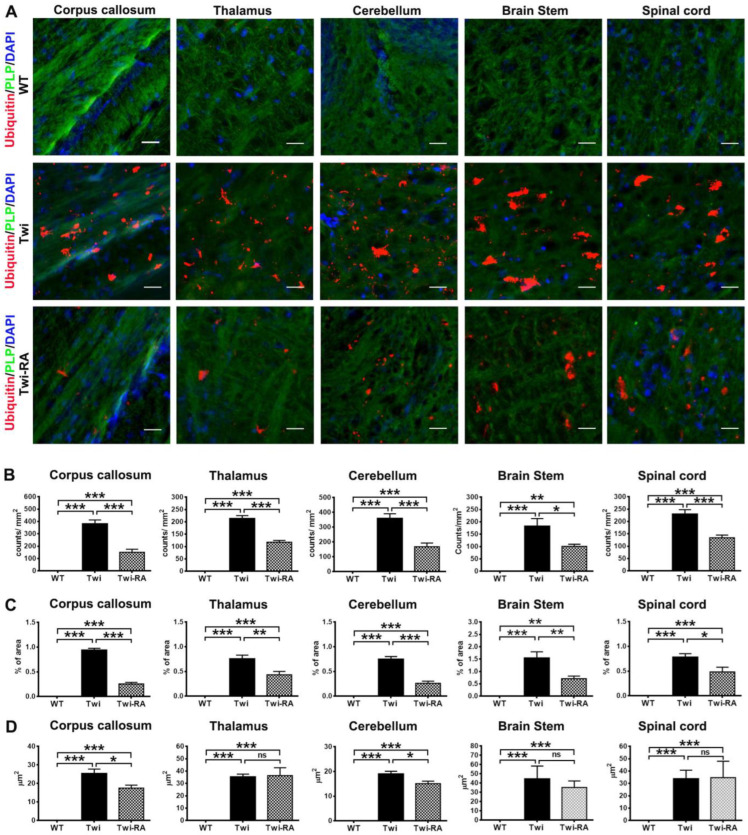
Accumulation of ubiquitin aggregates alleviated by rapamycin. (**A**) Representative images of ubiquitin (red), PLP (green), and nuclei (DAPI) immunostaining in different regions of the brain of wild-type (WT), untreated twitcher (Twi), and rapamycin-treated twitcher (Twi-RA) mice at 35 days of age. Scale bars: 200 μm. Total amounts of ubiquitin aggregates per square millimeters (**B**), the percentage of area occupied by ubiquitin aggregates (**C**), and the average size of ubiquitin aggregates were quantitatively analyzed with ImageJ (**D**). Values are expressed as mean ± SEM and compared using One way ANOVA (Tukey post hoc test) (*n* = 5). Statistical significance, * *p* < 0.05, ** *p* < 0.01, *** *p* < 0.001, and ns (not significant).

The distribution of ubiquitin aggregates in the brain of experimental animals was illustrated with immunofluorescent staining (Figure 4A). In line with our previous studies, the distribution of ubiquitin aggregates in regions of the brain was consistent with that of p62 aggregates in twitcher mice, where abundant ubiquitin aggregates were observed in the spinal cord, brain stem, cerebellum arbor vitae, thalamus, and corpus callosum (Figure 4A). Administration of rapamycin to twitcher mice did not alter the distribution of ubiquitin aggregates in the brain compared to untreated twitcher mice. Nonetheless, rapamycin treated twitcher mice showed a reduction of ubiquitin aggregates in comparison with that of untreated twitcher mice using qualitative assessment (Figure 4A). Using quantitative analysis, the total number of particles of ubiquitin aggregates in rapamycin-treated twitcher mice was significantly reduced to nearly 50% of that of untreated twitcher mice (Figure 4B). In line with this result, administration of rapamycin to twitcher mice significantly reduced the percentage of area occupied by ubiquitin aggregates in comparison with that of untreated twitcher mice (Figure 4C). The average size of ubiquitin aggregates was significantly reduced in corpus callosum and cerebellum of rapamycin-treated twitcher mice compared to untreated twitcher mice (Figure 4D). Overall, these findings indicated that rapamycin decreases the accumulation of insoluble ubiquitin and the formation of ubiquitin aggregates in twitcher mice.

### 3.3. Alleviation of Reactive Astrocytes

**Figure 5:**
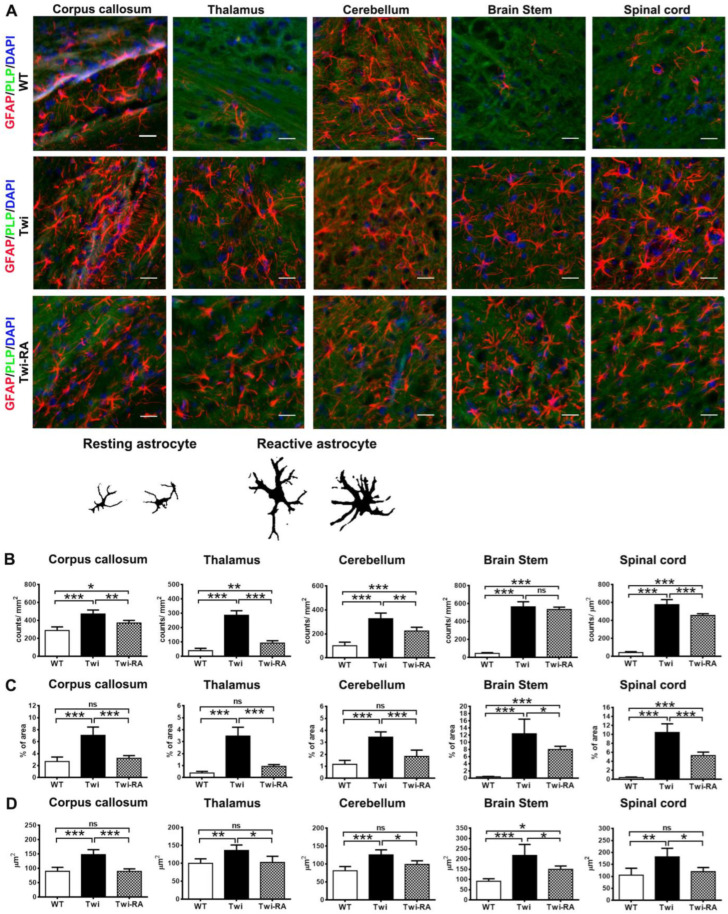
Rapamycin alleviates activation of astrocytes. (**A**) Representative images of GFAP (red), PLP (green), and nuclei (DAPI) immunostaining in different regions of the brain of wild-type (WT), untreated twitcher (Twi), and rapamycin-treated twitcher (Twi-RA) mice at 35 days of age. Scale bars: 25 μm. (**B**) Representative images of resting and reactive astrocytes. Total amounts of astrocytes per square millimeters (**B**), the percentage of area occupied by astrocytes (**C**), and the average size of astrocytes (**D**) were quantitatively analyzed with ImageJ. Values are expressed as mean ± SEM and compared using One way ANOVA (Tukey post hoc test) (*n* = 5). Statistical significance, * *p* < 0.05, ** *p* < 0.01, *** *p* < 0.001, and ns (not significant).

Widespread astrocytosis is the pathological hallmark of GLD [31]. Herein, distinct morphologies of astrocytes were clearly recognized by immunohistochemically labeled GFAP in the brains of wild-type and twitcher mice (*n* = 5 in each group) (Figure 5A). Resting astrocytes exhibit slender soma with short, thin, and ramified cellular processes, while reactive astrocytes are characterized by hypertrophic soma and stellate processes in response to pathological situation (Figure 5B). On day 35, the corpus callosum, cerebellar white matter, brain stem, and spinal cord of twitcher mice showed many reactive astrocytes with hypertrophic soma and thick stellate processes (Figure 5A). Of note, more resting astrocytes and less hypertrophic reactive astrocytes were observed in the brains of rapamycin-treated twitcher mice (*n* = 5) comparing to those of untreated twitcher mice, though anatomical distribution of astrocytes was similar in both groups (Figure 5A). Meanwhile, the brains of wild-type mice demonstrated sparse distribution of resting astrocytes with small soma and thin cellular processes in the corpus callosum and cerebellar white matter (Figure 5A).

The total amount and surface area of astrocytes were quantitatively analyzed and compared between groups. It was shown that the corpus callosum, thalamus, cerebellum, brain stem, and spinal cord of both untreated and rapamycin-treated twitcher mice demonstrated a remarkable increase in astrocytes comparing to those of wild-type mice (Figure 5B). Administration of rapamycin reduced the amounts of astrocytes in the corpus callosum, thalamus, cerebellum, and spinal cord of treated twitcher mice comparing to untreated twitcher mice (Figure 5B). The effects of rapamycin on the percentage of area occupied by astrocytes and the average size of astrocytes were also examined. In line with the increase of astrocytes, the percentage of area occupied by astrocytes and the average size of astrocytes in the brains of untreated twitcher mice was significantly increased in comparison with that of wild-type mice (Figure 5C). Importantly, rapamycin-treated twitcher mice showed a significant reduction in the percentage of area occupied by astrocytes compared to untreated twitcher mice, suggesting the reduction of astrocytosis (Figure 5C). Furthermore, it was shown that the average size of astrocytes was increased significantly in untreated twitcher mice in comparison with that of wild-type mice (Figure 5D), suggesting the activation of astrocytes into reactive astrocytes. Of note, rapamycin reduced the average size of astrocytes significantly in treated twitcher mice in comparison with untreated twitcher mice (Figure 5D). These results indicate that enhancement of autophagy within twitcher mice is associated with attenuation in activation of reactive astrocytes.

### 3.4. Alleviation of Microglia Activation

**Figure 6:**
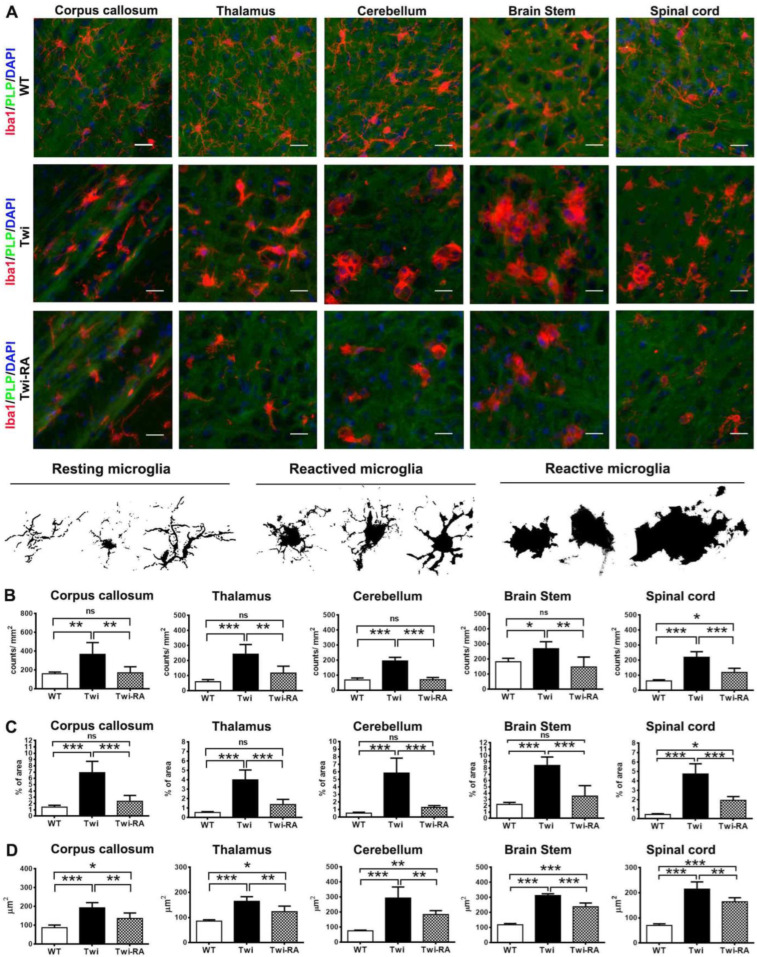
Amelioration of microglia activation by rapamycin. (**A**) Representative images of distribution of microglia in different regions of the wild-type (WT), untreated twitcher (Twi), and rapamycin-treated twitcher (Twi-RA) mice brain at ages 35 days. The brain was immuno-stained with anti-Iba1 (in red) and anti-PLP (in green), respectively, and nuclei were counterstained with DAPI (in blue). Scale bar: 25 μm. (**B**) Representative images of microglia activation phenotype. Total amounts of microglia per square millimeters (**B**), the percentage of area occupied by microglia (**C**), and the average size of microglia (**D**) were quantitatively analyzed with ImageJ. Values are expressed as mean ± SEM and compared using One way ANOVA (Tukey post hoc test) (*n* = 5). Statistical significance, * *p* < 0.05, ** *p* < 0.01, *** *p* < 0.001, and ns (not significant).

Early activation of microglia contributing to the formation of globoid cells represents the hallmarks of GLD pathology and plays a pivotal role in the mechanism of demyelination [27,32]. Herein, microglia were recognized by immunofluorescent staining (*n* = 5 in each group) (Figure 6A). Microglia phenotypes resting, activated, and reactive microglia were characterized by their distinctive morphology (Figure 6A). The resting microglia phenotype is typically characterized by small soma and ramified processes. After activation, resting microglia undergo morphological changes characterizing by hypertrophic cell bodies with bush, long, or short thickened processes [28]. Furthermore, the activated microglia are polarized into reactive phenotype characterized by ameboid or enlarged multinucleated phagocytes (globoid cells) without cellular processes in the CNS of GLD [28]. In this study, the resting microglia comprised most of the microglia observed in the brains of wild-type mice, while the reactive microglia with ameboid-like and muti-nucleus giant globoid cells were increased in the brains, especially the brain stem and spinal cord, of untreated twitcher mice (Figure 6A).

As expected, the reactive microglia were not observed in the brains of wild-type mice under normal conditions. Conversely, in the brains of untreated twitcher mice, there was an increase in activated phenotype and reactive ameboid microglia and multinucleated globoid cells, especially in the brain stem and spinal cord (Figure 6A). Of note, rapamycin treatment in twitcher mice alleviated the extent of resting and reactive microglia compared to untreated twitcher mice (Figure 6A). Furthermore, in line with these qualitative findings, quantitative analysis of Iba1-positive microglia from the corpus callosum, thalamus, cerebellum, brain stem, and spinal cord of wild-type, untreated twitcher, and rapamycin-treated twitcher mice revealed evident differences in the cell counts and surface area of microglia between these groups. As anticipated, untreated twitcher mice showed significantly increased counts of microglia compared to wild-type mice, suggesting the activation of microgliosis (Figure 6B). Intriguingly, the administration of rapamycin to twitcher mice significantly ameliorated the extent of microglia cells in the corpus callosum, thalamus, cerebellum, and brain stem, compared to untreated twitcher mice, to levels indistinguishable from those of wild-type mice (Figure 6B). Furthermore, in quantitative analysis of the percentage of area occupied by microglia, untreated twitcher mice demonstrated significant elevation of the total surface area of microglia compared to wild-type mice (Figure 6C). In contrast to this, and in line with our expectations, rapamycin significantly reduced the percentage of area occupied by microglia in twitcher mice compared to untreated twitcher mice, suggesting the amelioration of microgliosis (Figure 6C). Moreover, the percentage of area occupied by microglia in the corpus callosum, thalamus, cerebellum, and brain stem showed no significant difference between wild-type mice and rapamycin-treated twitcher mice (Figure 6C). Following this, the average size of microglia was calculated. In line with the qualitative analysis of Iba1-positive microglia in the corpus callosum, thalamus, cerebellum, brain stem, and spinal cord both untreated and rapamycin-treated twitcher mice showed an increase in the average size of microglia compared to wild-type mice, suggesting the activation of resting microglia into activated and reactive microglia (Figure 6D). Of note, rapamycin administration significantly reduced the surface area per microglia in twitcher mice compared to untreated twitcher mice (Figure 6D). This finding was compatible with the decrease in reactive ameboid microglia and multinucleated globoid cells in rapamycin-treated twitcher mice in qualitative analysis. Overall, our findings validate microgliosis with activation of microglia and increase in globoid cells in twitcher mice, which is attenuated significantly by rapamycin.

### 3.5. Rapamycin Preserves Cortical Myeloarchitecture

**Figure 7:**
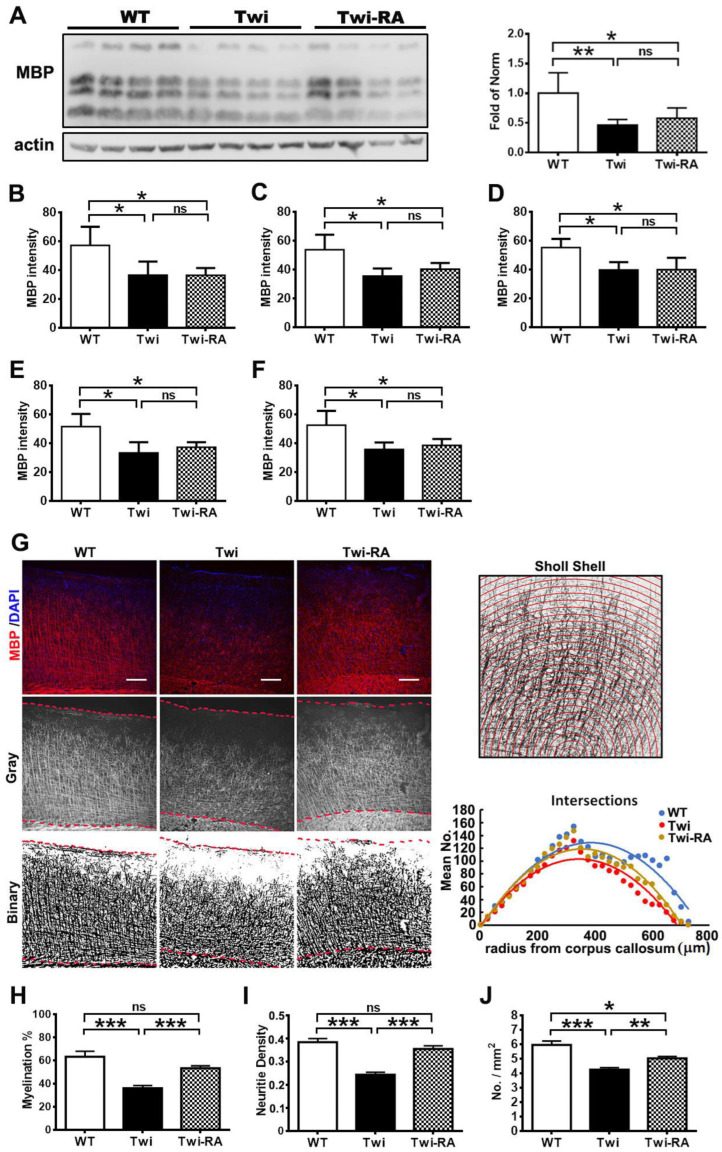
Rapamycin preserves myelin architecture. (**A**) Western blot analysis of MBP expression in brain stem of wild-type (WT), untreated twitcher (Twi), and rapamycin-treated twitcher (RM) mice brains at ages 35 days. Band intensities were measured by densitometry, normalized to actin expression, and are relative to wild-type mice. Values are expressed as mean ± SD and compared using One way ANOVA (Tukey post hoc test). (*n* = 4). The brain was immuno-stained with anti-MBP. Myelination of MBP fluorescence intensity in corpus callosum (**B**), thalamus (**C**), cerebellum (**D**), brain stem (**E**), and spinal cord (**F**) was quantitatively analyzed with ImageJ. Cortical myelination (dash area) was quantitatively analyzed with ImageJ. (**G**) Illustration of cortical myelination in MBP fluorescence (red), gray, and binary images, respectively. The proportion of myelination (**H**) and neuritis density (**I**) in field of cortex were quantified by measuring the MBP gray value and binary images, respectively. Microstructure complexity of cortical myelination was assessed by Sholl analysis and the numbers of intersections for concentric circles was plotted (**G**) and summed (**J**) for comparison. Values are expressed as mean ± SEM and compared using One way ANOVA (Tukey post hoc test) (*n* = 5). Statistical significance, * *p* < 0.05, ** *p* < 0.01, *** *p* < 0.001, and ns (not significant). Scale bar: 200 μm.

As mentioned above, our results demonstrated the therapeutic efficacy of rapamycin in the reduction in cytoplasmic aggregates in the CNS of twitcher mice. To further investigate whether the reduction in cytoplasmic aggregates showed potential action to prevent demyelination in GLD, we examined myelin status in the brains of untreated and rapamycin-treated twitcher mice compared to wild-type mice (*n* = 4 in each group). As anticipated, western blot analysis demonstrated a significant decrease in the expression of MBP in the brain stem of untreated twitcher mice compared to their wild-type littermates (Figure 7A). The administration of rapamycin to twitcher mice increased levels of MBP expression moderately compared to untreated twitcher mice, and did not return the MBP expression to the wild-type level. Furthermore, analysis of myelination by quantitation of MBP immuno-fluorescence intensity in the corpus callosum (Figure 7B), thalamus (Figure 7C), cerebellum (Figure 7D), brain stem (Figure 7E), and spinal cord (Figure 7F) did not demonstrate significant difference between rapamycin-treated and untreated twitcher mice.

To further investigate the effects of rapamycin on white matter structure, we examined the myelination status in the cortex, where ascending and descending myelinated axons with fiber crossing or complexity extend radially and parallelly from white matter into the cortex up to the external lamina allowing for an estimation of neurites dispersion, orientation, density, and microstructural organization [29]. Herein, fluorescent images of MBP-labelled brain slices were converted to gray and binary images, respectively, using the ImageJ software for quantitative analysis (Figure 7G). As anticipated, the proportion of cortical myelination was decreased significantly in untreated twitcher mice compared to wild-type littermates (*n* = 5 in each group) (Figure 7H). Whereas administration of rapamycin to twitcher mice preserved the proportion of cortical myelination comparable with that of wild-type mice and significantly more than that of untreated twitcher mice (Figure 7H). Furthermore, in accordance with the results of cortical myelination, the density of cortical neurites of untreated twitcher mice was reduced significantly compared to wild-type mice and rapamycin-treated twitcher mice (Figure 7I). Of note, the density of cortical neurites in rapamycin-treated twitcher mice was indistinguishable from that of wild-type mice (Figure 7I). To further understand if rapamycin preserves the integrity of the cortical neural network, neurites morphology was analyzed by Sholl analysis (Figure 7G). The number of intersections per concentric shell was recorded and added up for quantitative analysis. The total number of intersections in the cortical field of both untreated and rapamycin-treated twitcher mice were significantly reduced compared to wild-type mice (Figure 7J). Interestingly, rapamycin-treated twitcher mice demonstrated significantly more intersections in cortex compared to untreated twitcher mice (Figure 7J). Overall, these results suggest that rapamycin treatment preserved the cortical myeloarchitecture in the brains of twitcher mice.

### 3.6. Body Weight Was Not Altered by Rapamycin

**Figure 8:**
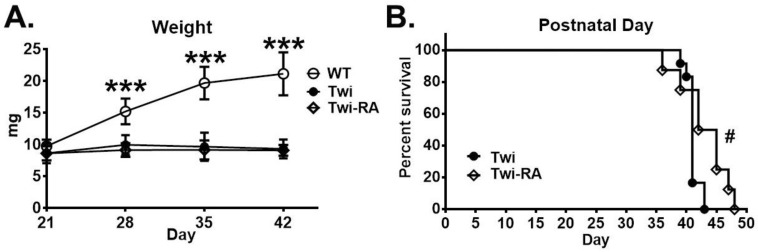
Rapamycin prolonged lifespan in twitcher mice. (**A**) Body weight of wild-type (WT; *n* = 12) mice, untreated twitcher (Twi; *n* = 12), and rapamycin-treated twitcher (Twi-RA; *n* = 8) mice was measured every 7 days commencing at 21 days of age. Values are expressed as mean ± SD and compared using One way ANOVA (Tukey post hoc test). Statistical significance, *** *p* < 0.001 (comparing to WT). (**B**) Kaplan–Meier curve showing increased lifespan in rapamycin-treated (Twi-RA; *n* = 8) mice compared to untreated twitcher (Twi; *n* = 12) mice. **#** *p* < 0.05 (Twi-RA versus Twi using Student’s *t* test).

The body weight was measured in experimental and control animal groups every 7 days commencing at 21 days of age (Figure 8A) to determine the therapeutic efficacy upon phenotypic manifestation. Our results demonstrated that both untreated twitcher mice and rapamycin-treated twitcher mice showed lower body weight compared to wild-type mice at each time point. Herein, the weight of rapamycin-treated mice was indistinguishable from that of untreated twitcher mice.

### 3.7. Prolonged Lifespan by Rapamycin

Both the untreated and rapamycin-treated twitcher mice were monitored closely until their moribund stage and the life span was recorded accordingly. A plot of the Kaplan–Meier survival curve was graphed in accordance with the probability of survival for untreated and rapamycin-treated twitcher mice, respectively (Figure 8B). The median life of untreated twitcher mice was 41 days (range: 40–43 days), while rapamycin-treated twitcher mice had a median lifespan of 43.5 days (range: 39–48 days). It demonstrated a moderate but significant increase in lifespan for rapamycin-treated twitcher mice compared to untreated twitcher mice.

## 4. Discussion

Several pieces of evidence indicated that disturbed autophagy plays a pivotal role in the pathogenesis of white matter disorders and therapeutics targeting autophagy may be a promising strategy [33]. Our previous studies have demonstrated the fundamental aspect of GLD in that both impaired autophagy and dysfunction of UPS lead to the broad deposition of p62- and ubiquitin-aggregates resulting in the death of oligodendrocytes in the central nervous system [14]. Following our previous findings, we further demonstrate the efficacy of mTOR inhibitor rapamycin in the amelioration of the pathogenesis in GLD by reduction of protein aggregates, amelioration of neuroinflammation, and the preservation of the myeloarchitecture of cortical neurites in twitcher mice. It is noted that our results agree with previous studies using rapamycin in different diseases associated with intracellular aggregates and further broaden the scope of rapamycin as a potential therapeutic for LSD and white matter disorders.

Both autophagy and UPS work coordinately to maintain cellular homeostasis. The short-lived, unfolded, and misfolded proteins are mainly degraded in the UPS. On the other hand, insoluble large protein aggregates, long-lived proteins, and dysfunctional/damaged organelles are degraded in the lysosomal system of autophagy. Numerous studies have linked the alternation of proteostasis to the pathogenesis of neurodegenerative diseases, and the modulation of proteolytic machinery to restore or enhance the degradation pathway is considered a promising therapeutic approach for amelioration of the pathogenesis [23,24,25]. Our previous studies demonstrated co-deposition of p62 and ubiquitin into aggregates [14]. In present studies, the total number of particles of p62-aggregates and ubiquitin-aggregates were remarkably reduced to 50% and 55% of that of untreated twitcher mice, respectively, by rapamycin administration. Herein, our results demonstrate that rapamycin reduces the burden of p62- and ubiquitin-aggregates in the brain of twitcher mice, concomitant with the diminished levels of phosphor-S6 protein, suggesting the degradation of aberrant aggregates via inhibition of mTOR signaling pathway. Of note, the turnover of LC3-II, levels of insoluble p62, and area of p62 aggregates were altered moderately without statistical difference, albeit the total particles of p62 aggregates were significantly reduced with rapamycin treatment. This limited efficacy of autophagic degradation may be partially explained by the psychosine toxicity induced disruption of lysosomal function during the progression of disease [34]. Meanwhile, the decreased accumulation of insoluble ubiquitin expression and aggregates after rapamycin treatment indicates an increased degradation of insoluble polyubiquitylated protein via UPS preventing the formation and deposition of insoluble protein aggregates in twitcher mice brains. It has been noted that rapamycin-mediated mTOR inhibition increases both autophagy and proteasome capacity in yeast and mammalian cells [35,36,37]. In line with these notes, inhibiting mTOR with rapamycin rapidly increases the degradation of protein aggregates by stimulating both autophagy and proteasome-mediated proteolysis [21]. These studies and our results are in line with the prevailing view that mTORC1 inhibition is directly linked to the clearance of protein aggregates.

Neuroinflammation is a pathologic hallmark of GLD. It has been noted that microgliosis precedes activation of astrocytes in the hindbrain of twitcher mice as early as 2 weeks of age [28,32]. At 3 weeks of age, microglia activation concurrent with aggregates of amoeboid microglia distributes diffusely in the hindbrains of twitcher mice, and it progresses to both the forebrain and hindbrain at 5 weeks of age, where microglia aggregates form multinucleated globoid cells as the pathological hallmark of GLD [28,32]. Astrocytes are first activated around glia nodules at 3 weeks of age and distribute diffusely throughout the twitcher brain at 5 weeks of age [28]. The activation of astrocytes and microglia has a detrimental effect on the disease progression of GLD. In agreement with previous studies, our results demonstrated the abundant distribution of reactive astrocytes/microglia, amoeboid microglia, and multinucleated globoid cells in the hindbrains of twitcher mice at 35 days of age, while rapamycin attenuated the activation of astrocytes/microglia and the polarization of activated microglia to amoeboid microglia/globoid cells in the hindbrain of treated twitcher mice. The therapeutic effects of rapamycin upon the alleviation of neuroinflammation may be mainly attributed to the reduction of disease protein aggregates via enhancement of degradation. It has been noted that progressive deposition of insoluble protein aggregates in CNS induces reactive microglia and chronic microgliosis contributing to deterioration in neurodegenerative diseases, while enhancement of degradation reduces deposition of protein aggregates, diminishes microglia activation, and prevents neuronal cell death [38,39,40,41]. In turn, reduction of microglia activation and its secretion of cytokine/chemokine attenuates astrocyte proliferation leading to improvement of neuronal survival and axonogenesis [42]. Of note, it has been demonstrated that rapamycin attenuates immune inflammation and reactive astrocytes by directly modulating mTOR-Akt-NF-*κ*B cascade, glutamate transporter, and IL-6 expression in astrocytes [43]. Together, alleviation of reactive microglia and astrocytes after rapamycin treatment could be attributed to both enhanced clearances of protein aggregates and direct anti-inflammation effects on microglia and astrocytes. Herein, our findings implicate the beneficial effects of rapamycin in the treatment of leukodystrophies via modulation of disease protein degradation and neuro-inflammation.

It has been shown that protein aggregates in oligodendrocytes exert cytotoxicity interfering with cellular processes, which causes cell death and subsequent demyelination exacerbating the progression of neurodegenerative disease [44,45]. In line with this note, our previous studies revealed that psychosine impairing autophagy and UPS leads to the accumulation of cytoplasmic p62- and ubiquitin-aggregates in oligodendrocytes attenuating the cell viability and mitochondrial function, which induces global demyelination in GLD [14]. Following these findings, enhanced degradation for removal and clearance of disease protein aggregates is expected to improve demyelination in this study. As anticipated, rapamycin attenuates deposition of ubiquitin-aggregates and total particles of p62 aggregates in twitcher mice brains. These findings coincide with our observation that maintenance of the cortical myeloarchitecture of rapamycin-treated twitcher mice compared to untreated twitcher mice suggests the alleviation of demyelination and preservation of myelinated structure after enhanced clearance of disease protein aggregates. In support of this, the reduction of ameboid microglia and globoid cells in rapamycin-treated twitcher mice brains suggests a result of alleviation of myelin loss, given that activated microglia are transformed into ameboid microglia and globoid cells after phagocytosis of damaged myelin debris [27]. In concurrence with these data, rapamycin significantly preserves the myelinated region, fiber density, and myeloarchitecture in the cerebral cortex in comparison with that of untreated twitcher mice. This therapeutic effect of rapamycin in the maintenance of myelination integrity by enhanced clearance of misfolded protein aggregates has been demonstrated in other demyelinating diseases. In both in vivo and in vitro models of Charcot–Marie–Tooth disease, which is characterized by progressive demyelination of peripheral nerves and associated neuromuscular deficits, the activation of degradation by rapamycin decreases the accumulation of aberrant protein aggregates within neuropathic Schwann cells, improving the myelination while inhibition of proteolytic degradation abolishes the effect of rapamycin against demyelination [46,47,48]. Additionally, the therapeutic effects of rapamycin on alleviation of demyelination can be attributed in part to the direct impact on myelinating glia through regulating cell growth and differentiation. It has been noted that rapamycin restores the axonal domain organization, development, and proliferation of oligodendrocytes through the upregulation of proteolytic degradation, leading to the prevention of demyelination and promotion of remyelination [49,50]. Moreover, the reduction of neuroinflammation through the immunomodulatory properties of rapamycin contributes collaboratively to the alleviation of myelin loss and preservation of myeline integrity, given that astrocytosis and microgliosis exacerbate the progressive demyelination and morbidity in leukodystrophies [32,51,52].

In this context, our study first shows small molecule drugs reducing disease protein aggregates, attenuating neuroinflammation, preserving myelination, and prolonging life span through inhibition of mTOR signaling and enhanced clearance of protein aggregates. Although rapamycin alleviates the pathogenesis, the lifespan is extended moderately in treated twitcher mice. This is mainly attributed to the deficiency of GALC enzyme activity and the progressive accumulation of psychosine, both of which need GALC-supplying therapy. As the psychosine cytotoxicity emerges at the neonatal life-stage leading to the accumulation of the misfolded protein during progression of the disease and premature death after an average life of 39 days, the introduction of rapamycin at 21 days of age, when the clinical phenotype begins to occur, may not be able to degrade previously accumulated misfolded proteins, suggesting the need for early interventions. Furthermore, the progressive accumulation of psychosine impairs the lysosomes of autophagy cargo and disrupts the recruitment of key components of the autophagy machinery, leading to vulnerability of autophagy capacity and highlighting the complementary therapy to reduce toxic substrate accumulation [19,34,53]. However, the substrate reduction therapy reduces levels of psychosine partially and has modest effects on pathogenesis and the life span of twitcher mice [53]. Likewise, cellular therapy and gene therapy providing GALC enzyme replacement and immuno-modulation have demonstrated rescue of GALC deficiency and amelioration of demyelination and inflammation concomitant with a significant prolongation of lifespan in twitcher mice, albeit the longevity of surviving animals still did not reach the lifespan of wild-type mice and the pathological remarks were still manifested progressively [54]. These findings and our data implicate the complex mechanism of pathogenesis for GLD and highlight the combination therapies targeting both the primary and secondary patho-mechanism. Following this, the combination of gene therapy, cellular therapy, and enzyme replacement therapy has shown synergic efficacy in slowing the deterioration of phenotype and the progression of pathogenesis in twitcher mice, compared to either treatment regimen alone, albeit complete rescue remains a challenge [55]. Herein, our data demonstrate the efficacy of rapamycin in the treatment of twitcher mice and suggest rapamycin as a promising complementary regimen for combination therapies to obtain a thorough correction of pathogenesis in GLD.

## 5. Conclusions

In summary, we first show the efficacy of small molecules rapamycin in the preservation of cortical myeloarchitecture and the alleviation of astrocytes and microglia activation by inhibition of mTOR signaling and enhanced clearance of protein aggregates in twitcher mice. In line with this, the pharmacological enhancement of degradation of misfolded protein aggregates offers promise for the complementary regimen in combination therapies for GLD. In addition to the treatment of neurodegenerative disease with proteinopathies, our findings have broadened the scope for rapamycin as a potential therapeutic for LSD and leukodystrophies associated with intracellular aggregation.

## Data Availability

The datasets used and/or analyzed during the current study available from the corresponding author on reasonable request.
